# Cross-sectional associations of objectively assessed sleep duration with physical activity, BMI and television viewing in German primary school children

**DOI:** 10.1186/s12887-019-1429-3

**Published:** 2019-02-11

**Authors:** Susanne Kobel, Olivia Wartha, Jens Dreyhaupt, Sarah Kettner, Jürgen M. Steinacker

**Affiliations:** 1 Division of Sports and Rehabilitation Medicine, Department of Internal Medicine II, Ulm University Medical Centre, Frauensteige 6, Haus 58/33, D-89075 Ulm, Germany; 2 Institute of Epidemiology and Medical Biometry, Ulm University, Schwabstr. 13, D-89075 Ulm, Germany; 3 Division of Sports and Rehabilitation Medicine, Department of Internal Medicine II, Ulm University Medical Centre, Leimgrubenweg 14, D-89075 Ulm, Germany

**Keywords:** MVPA, Body composition, TV watching, Accelerometer, Migration

## Abstract

**Background:**

The global incidence of overweight and obesity has increased dramatically among children and adolescents over the past decades. Insufficient sleep duration and physical inactivity are known risk factors for overweight and obesity in children. To engage children in a healthier lifestyle knowledge about associations of sleep duration and behavioural aspects in children are vital. Therefore, this study investigated the mentioned associations in German primary school children.

**Methods:**

Data of 308 first and second graders (7.1 ± 0.6 years) was used; children’s anthropometric data were taken during a school visit. Children’s physical activity (PA) and sleep duration were assessed objectively (Actiheart©, CamNtech Ltd., Cambridge, UK); children’s daily television time and socio-demographic data were collected via parental questionnaire. Linear mixed-effects regression models as well as logistic regressions were used to determine associations of PA, television viewing, age, gender, BMI z-scores and socio-economic variables on sleep duration.

**Results:**

In linear regression models young age and not having a migration background were significantly associated with long sleep duration (*p* < 0.001). In logistic regressions, long night time sleep (≥10:08 h; compared to medium and short sleep duration) was significantly associated with not reaching the PA guideline (OR 0.60 [0.36;0.99]), daily television viewing of less than one hour (OR 0.44 [0.24;0.80]), young age (OR 0.38 [0.21;067]), a high parental education level (OR 0.52 [0.27;0.99]) and the lack of migration background (OR 0.21 [0.10;0.48]). However, if controlling for age, gender, parental education level and migration background, reaching the PA guideline stayed no longer significantly associated with a tertiary sleep level.

**Conclusions:**

Children in the highest sleep category showed a negative association with reaching the PA guideline and a positive association with daily television viewing. This therefore adds to previously primarily subjectively assessed associations of sleep and risk factors for obesity (related behaviours) with a detailed insight based on objective data. Hence, interventions trying to decrease children’s BMI and television viewing should also aim at extending children’s night-time sleep and inform parents about the importance of sufficient sleep during childhood.

**Trial registration:**

DRKS-ID: DRKS00000494.

## Background

The emergence of overweight and obesity has become a global health problem that no longer affects only wealthier industrialised countries, but is also increasingly spreading in developing countries [1]. The increase in body weight can lead to serious health consequences such as cardiovascular diseases [2], metabolic disorders, such as diabetes mellitus [3], psychological and motor developmental delays [4], and emotional stress [5].

The global incidence of overweight and obesity has also increased dramatically among children and adolescents over the past 30 years [6]. If this trend continues, experts predict 11% of overweight children worldwide in 2025 [7], already in 2016, over 41 million children under the age of five are estimated to be overweight [8]. Since children are dependent on their parent’s behaviour, especially their nutrition and physical activity behaviour [9, 10], it is important to introduce children to a healthy lifestyle as early as possible in order to embed healthy habits into their daily life [11]. Reasons for children’s energy unbalance are increased energy intake, often caused by the consumption of sugary drinks as well as too little physical activity and too high screen media use [12]. This trend is particularly evident in children starting school [13, 14].

In order to counteract this and since regular physical activity has shown to have numerous benefits to children’s health [15, 16] the World Health Organisation advocates a daily amount of 60 min of moderate to vigorous physical activity (MVPA) for children and adolescents [17]. In spite of this, many children are not sufficiently active enough to benefit their health. Depending on assessment method, interpretation, sample and region, between 87% and 3–5% of European youth is considered sufficiently active [18]. In the US, nearly 50% of children fail to meet the minimum requirement for daily physical activity [19] and less than a third and a quarter of English boys and girls, respectively, between two and 15 years meet the recommended 60 min or more of MVPA a day [20]. In Germany, less than 20% of primary school children are sufficiently active [21].

However, not only physical inactivity, but also insufficient sleep duration is a recognised risk factor for children’s health [22–25], which importance grows from the time of school entry [26]. Current recommendations, considering children’s overall well-being as well as cognitive, emotional and physical health, advocate nine to eleven hours of sleep per day for 6- to 13-year-olds [27]. Based on those recommendations, internationally, numerous studies investigated the influence of sleep on obesity and obesity-related behaviours in children [28, 29]. In German as well as Chinese primary school children for instance, a daily sleep duration of 10 h or less was significantly associated to being overweight or obese [26, 28]. Similarly, boys who slept less than 10 h per night showed an increased risk of higher fasting blood glucose [29] and a large review found consistent associations between short sleep duration and more screen media use [30].

Further, there have been many attempts to find associations between children’s night-time sleep duration and their physical activity behaviour during the day – especially considering the physical activity guideline of 60 min of moderate to vigorous physical activity (MVPA) daily [17] – although so far with inconsistent findings. Previous research has shown that children’s sleep duration was positively [31], negatively [32, 33], or not at all [24, 34, 35] related to their physical activity behaviour on the next day. The reasons for these inconsistencies remain unclear; however, many of the studies examining sleep duration base their data on subjective assessments, such as questionnaires or proxy reports, which have known disadvantages and partially invalid for assessing sleep duration [36]. Especially in Germany, there is a lack of data on objectively assessed children’s sleep duration and physical activity. Hence, this study investigated associations of objectively assessed sleep duration with physical activity behaviour, body composition and television viewing in German primary school children.

## Methods

### Study population

Baseline data of a sub-sample of 308 first and second graders of 32 primary schools (7.1 ± 0.6 years) taking part in the so-called Baden-Württemberg study in south-west Germany [37], was used. Details on its study design and protocol as well as the recruiting process can be found elsewhere [38]. Parents provided their written informed consent to take part in the study and a separate consent for their children to wear a multi-sensor device assessing sleep and physical activity objectively for six consecutive days; children gave their verbal assent on the day of assessment. The study was approved by the university’s ethics committee (application no. 126/10) and conducted in accordance with the declaration of Helsinki.

### Anthropometric measurements

Children’s anthropometric data (height (cm) and body weight (kg)) were taken by trained staff using a stadiometer and calibrated electronic scales (Seca 213 and Seca 826, respectively, Seca Weighing and Measuring Systems, Hamburg, Germany). Subsequently, children’s body mass index (BMI) was calculated (kg/m^2^) and converted to BMI z-scores [39].

### Sleep and physical activity assessment

Children’s physical activity and sleep duration were assessed objectively by a chest-worn multi-sensor device (Actiheart©, CamNtech Ltd., Cambridge, UK) which measures uniaxial acceleration in combination with heart rate. Validity of the device in children has been established previously [40]. Although this technique is primarily used to assess physical activity and sedentary behaviour, it has found increasing use in the field of sleep medicine in recent years [41, 42].

The device’s recording interval was set to 15 s and participants wore it for six consecutive days and nights (for further details see [43]). To be included in the physical activity analyses, at least three days (including at least one weekend day) of valid data of more than 10 h were required (as recommended by Trost et al. [44]). First and last recording days were excluded from the analysis to antagonise a novelty factor on the first day, whereas the last day never showed 10 h of recording. In order to show valid sleep data, at least three nights, including at least one at the weekend were necessary (as recommended by the American Academy of Sleep Medicine [45]). Individual sleep duration was based on the children’s individual heart rate variability in combination with assessed inactivity, which allows the determination of the exact time of falling asleep and awaking. Both of which was identified by two independent experts and subsequently calculated in hours per night-time sleep.

Children’s physical activity levels were determined on the basis of energy expenditure (MET) predicted by Actiheart©‘s captive software (Version 4.0.129), taking into account participant’s age, height, body weight and gender in addition to the assessed heart rate and movement counts. Physical activity was then classified into sedentary (< 1.5 MET), light (1.5–3 MET), moderate (> 3–6 MET), and vigorous (> 6 MET) as well as MVPA (> 3 MET) for each 15 s recording interval [46]. In order to determine whether participants met the WHO physical activity guideline of 60 min of MVPA every single day [17], the days with valid data were extrapolated to a full week, using a ratio of 5:2 for weekdays and weekend days.

### Behavioural and socio-demographic data

Behavioural and socio-demographic data were collected using standardised and validated questions [47] in a parental questionnaire. Children’s daily television time was assessed for weekdays as well as weekend days on a categorical level. A ratio of 5:2 was used to determine daily television time. Parental education was based on the highest school education of either one parent or the single parent. Further, children were classified as having a migration background if at least one parent was born abroad or the child was spoken to in foreign language during the first three years of life.

### Statistical analysis

For logistic regressions, children’s sleep duration in hours and minutes was dichotomised by a primary/secondary and tertiary sleep level (total sleep duration of all children was split in three parts of equal frequency); therefore, tertiary sleep level was defined as an average sleep duration of 10 h and 8 min or more (compared to primary sleep level of 9:45 h or less or a secondary sleep level of between 9:46 h and 10:07 h). For logistic regression analyses, physical activity (in minutes per day) was dichotomised by reaching the WHO physical activity guideline of 60 min of MVPA every single day or not. Daily television viewing was dichotomised by one hour or more (median split as well as German recommendations [48]). For logistic regression analyses, children’s age was dichotomised at below seven years and seven years or more (median split). Socio-economic variables, such as parental education was dichotomised by primary/secondary and tertiary education level; i.e. having a high school degree or not; and children with at least one parent who was born abroad or were spoken to in foreign language during the first three years of life, were dichotomised as having a migration background.

Group differences between means were analysed with unpaired t-tests; linear regression and ANOVA were used to examine differences in BMI z-scores on the basis of children’s sleep duration. Linear mixed-effects regression models were used to determine associations of physical activity, television viewing, age, gender, BMI z-scores and socio-economic variables such as parental education and migration background on sleep duration, controlling for school effects. Logistic regressions were calculated for physical activity and television viewing, controlling for age, parental education and migration background.

Descriptive statistics for continuous variables are displayed in mean values and standard deviations. Categorical variables are described with absolute and relative frequencies. Statistical analyses were performed using SPSS Statistics 25 (SPSS Inc., Chicago, IL, US) and SAS (SAS Institute, Cary, NC, US) with a significance level set to α < 0.05.

## Results

### Sample characteristics

There are no differences between the here analysed subsample (*n* = 308) and the overall sample of the Baden-Württemberg study (*n* = 1947) with regard to the parameters age, gender, BMI z-scores, parental education level and migration background. A summary of participant’s anthropometric and socio-demographic characteristics as well as their physical activity level and television viewing is shown in Table 1.

**Table 1:** Descriptive characteristics of total sample, children with short/medium sleep duration (primary and secondary sleep level; < 10 h, 8 min) and children with long sleep duration (tertiary sleep level; ≥ 10 h, 8 min)

	Missing values	Total sample (*n* = 308)	Short/medium Sleep Duration (*n* = 202)	Long Sleep Duration (*n* = 106)
Gender (male); n (%)	0	151 (49.0)	106 (52.5)	45 (42.5)
Age (years)*; m ± SD	0	7.1 ± 0.6	7.2 ± 0.6	6.9 ± 0.6
Height (cm); m ± SD	1	123.6 ± 6.1	124.0 ± 6.1	122.7 ± 6.0
Weight (kg)*; m ± SD	1	24.6 ± 4.8	25.0 ± 4.8	23.8 ± 4.7
BMI z-scores; m ± SD	1	0.18 ± 1.11	0.25 ± 1.11	0.03 ± 1.10
Weight status	13			
Overweight; n (%)	13	36 (12.2)	26 (13.5)	10 (9.8)
Obese; n (%)	13	10 (3.4)	6 (3.1)	4 (3.9)
Sleep duration (h:min/night)*; m ± SD	0	9:58 (0:29)	9:42 (0:19)	10:29 (0:18)
MVPA Guideline reached*; n (%)	4	148 (48.7)	105 (52.8)	43 (41.0)
Daily MVPA (h:min/day)*, m ± SD	2	2:13 (0:58)	2:18 (0:59)	2:04 (0:55)
TV viewing of more than 1 h/day*; n (%)	41	150 (56.2)	108 (63.2)	42 (43.8)
Tertiary parental education level; n (%)	48	92 (35.4)	59 (36.2)	33 (34.0)
Migration background*; n (%)	41	71 (26.6)	60 (35.7)	11 (11.1)

Values are mean (m) ± SD or numbers (n) and percentages (%). Overweight and obese defined as per Cole et al. (2000); MVPA guideline reached = a minimum of 60 min of MVPA (moderate to vigorous physical activity) on all days of the week; TV viewing = television viewing; tertiary family education level = at least one parent has a high school degree; migration background = at least one parent was born abroad or the child was spoken to in foreign language during the first three years of life

*significant difference between children with short/medium and long sleep duration (*p* < 0.05)

### Sleep duration, physical activity and television viewing

On average, children slept 9:58 h (± 0:29) per night (ranging from 8 h 44 min to 12 h 2 min), with a significant difference between younger and older children (t = 5.72, *p* < 0.001) but no gender difference. Children of 6 years and younger (average age: 6.09 (± 0.17) years) for example slept on average 10:05 (± 0.33) hours, whereas children of 8 years and older (average age: 8.03 (± 0.37) years) slept 9:45 (± 0:27) hours. Also, children with migration background slept significantly less than children without migration background (9:46 (± 0:23) hours vs. 10:05 (± 0:30) hours, respectively; t = 4.85, *p* < 0.001).

The participating children spent on average 2:13 (± 0:58) hours per day in MVPA, with a significant gender difference (2:18 (± 0:59) hours for boys vs. 2:04 (± 0:55) hours for girls t = 9.75, *p* < 0.001). Also children spending less than 2 h in MVPA per day (median; average: 1:28 (± 0.02) hours) slept with 10:02 (± 0:02) hours significantly more than their more active counterparts (average: 2:58 (± 0.03) hours) with 9:55 (± 0:28) hours (t = 2.20, *p* < 0.028).

However, merely half of the children (48.7%) reached the physical activity guideline of 60 min of MVPA daily. Again, boys achieved this goal significantly more often than girls (68.7% vs. 29.2%, respectively; t = 7.46, *p* < 0.001). Age, migration background or parental education level were not associated with reaching the physical activity guideline, neither was sleep duration (10:01 (± 0:30) hours for children not reaching the guideline vs. 9:55 (± 0:27) hours for children reaching the guideline); age however was associated with daily MVPA (t = − 3.05, *p* < 0.003).

Children spent on average between 45 and 60 min per day watching television, with a significant difference between children from families with migration background and low parental education levels (t = − 3.54, *p* < 0.001 and t = 5.02, *p* < 0.001, respectively). Comparing children who watched television for one hour or more with those who watched television for less than one hour, a significant difference in sleep duration could be observed with children watching more television sleeping less (10:01 (± 0:39) hours vs. 9:50 (± 0:28) hours, t = 2.22, *p* < 0.027).

### Associations of BMI z-scores, physical activity and television viewing with sleep duration

The results from the linear mixed-effects regression models show significant associations between age and migration background with sleep duration (in hours per night) for the total sample and girls, with younger children and those without a migration background sleeping more (see Table 2) and significant associations between age, parental education level and migration background with sleep duration (in hours per night) for boys (see Table 2).

**Table 2:** Associations with daily sleep duration (in hours) displayed for all children, boys and girls (results from linear mixed-effects regression models)

	Total sample		Boys		Girls	
	Estimate [CI95%]	*p*	Estimate [CI95%]	*p*	Estimate [CI95%]	*p*
Daily MVPA [hours]	−0.01 [− 0.01;0.01]	0.423	0.01 [− 0.01;0.01]	0.883	− 0.01 [− 0.01;0.01]	0.157
TV viewing of more than 1 h/day [no vs. yes]	0.07 [− 0.10;0.24]	0.423	0.09 [− 0.15;0.34]	0.447	0.06 [− 0.18;0.31]	0.603
BMI z-score [−]	0.01 [−0.05;0.07]	0.726	−0.02 [− 0.10;0.06]	0.599	0.04 [− 0.04;0.13]	0.320
Age [years]	**−0.23 [− 0.32;-0.13]**	**0.001**	**− 0.23 [− 0.37;-0.08]**	**0.002**	**−0.24 [− 0.37;-0.11]**	**0.001**
Gender [female vs. male]	−0.10 [− 0.23;0.04]	0.151	N/A	N/A	N/A	N/A
Tertiary parental education level [no vs. yes]	0.08 [−0.05;0.19]	0.220	**0.20 [0.03;0.37]**	**0.021**	−0.06 [− 0.23;0.11]	0.501
Migration background [no vs. yes]	**0.32 [0.19;0.46]**	**0.001**	**0.23 [0.02;0.44]**	**0.035**	**0.37 [0.20;0.55]**	**0.001**

*CI* confidence interval, Daily MVPA = minutes of MVPA (moderate to vigorous physical activity) per day; TV viewing = television viewing; gender = male; tertiary family education level = at least one parent has a high school degree; migration background = at least one parent was born abroad or the child was spoken to in foreign language during the first three years of life

Bold = significant correlates (*p* < 0.05)

Weight status showed no significant difference in sleep duration with overweight and/or obese children sleeping on average 9:51 (± 0:28) hours whereas normal weight children slept 8 min longer (9:59 (± 0:30) hours). Also, a decrease of one minute of daily MVPA was associated with longer sleep duration, as was an increase of 0.01 points in BMI z-score (see Table 2), but none of those associations (BMI z-scores, physical activity (in hours per day) or television viewing) were statistically significant.

In the logistic regression analysis a sleep duration of 10 h and 8 min or more was associated (not significantly) with lower BMI percentiles z-scores (0.25 ± 1.11 vs. 0.03 ± 1.10, respectively; F = 0.93, *p* < 0.13), and significantly associated with lower daily television viewing (OR 0.45 [0.27;0.76], *p* ≤ 0.01) as well as negatively associated with reaching the physical activity guideline (OR 0.60 [0.36;0.99], *p* < 0.05). However, if controlling for age, gender, parental education level and migration background, reaching the physical activity guideline stayed no longer significantly associated with a tertiary sleep level (see Table 3).

**Table 3:** Odds Ratios for tertiary sleep level (results from multiple logistic regression models)

	Total sample		Boys		Girls	
	OR [CI95%]	*p*	OR [CI95%]	*p*	OR [CI95%]	*p*
MVPA Guideline reached	0.68 [0.39;1.20]	0.182	0.99 [0.39;2.51]	0.981	0.79 [0.33;1.89]	0.598
TV viewing of more than 1 h/day	**0.44 [0.24;0.80]**	**0.007**	0.46 [0.17;1.24]	0.123	**0.34 [0.15;0.77]**	**0.009**
Age	**0.38 [0.21;0.67]**	**0.001**	0.44 [0.18;1.05]	0.064	**0.27 [0.12;0.60]**	**0.001**
Tertiary parental education level	**0.52 [0.27;0.99]**	**0.046**	**0.25 [0.09;0.72]**	**0.010**	1.05 [0.44;2.50]	0.910
Migration background	**0.21 [0.10;0.48]**	**0.000**	**0.12 [0.02;0.56]**	**0.007**	**0.27 [0.10;0.72]**	**0.009**

*OR* Odds Ratio, *CI* confidence interval; MVPA guideline reached = a minimum of 60 min of MVPA (moderate to vigorous physical activity) on all days of the week; TV viewing = television viewing; tertiary family education level = at least one parent has a high school degree; migration background = at least one parent was born abroad or the child was spoken to in foreign language during the first three years of life

Bold = significant correlates (*p* < 0.05)

For the total sample, a long sleep duration was significantly positively associated with daily television viewing of less than one hour (OR 0.44 [0.24;0.80]), young age (OR 0.38 [0.21;0.67]), a high parental education level (OR 0.52 [0.27;0.99]) and the lack of migration background (OR 0.21 [0.10;0.48]; see Table 3).

If analysed separately for boys and girls, only having a migration background was significantly associated with a tertiary sleep level for both genders (OR 0.12 [0.02;0.56] and OR 0.27 [0.10;0.72], respectively). For boys, neither age nor television viewing stayed significantly associated with a long sleep duration, but parental education level still showed a significant association (OR 0.25 [0.09;0.72]). For girls on the other hand, young age and little television viewing were significant positive correlates of a tertiary sleep level (OR 0.27 [0.12;0.60] and OR 0.34 [0.15;0.77], respectively), however parental education lost its significance (see Table 3). Originally planned clustering for schools was neglected as no associations were found.

## Discussion

This study investigated associations of objectively assessed night-time sleep duration with daily MVPA, reaching the physical activity guideline of 60 min of MVPA per day, daily television viewing and body composition in the form of BMI z-scores in German primary school children. On average, children slept short of 10 h, with younger children and those without migration background sleeping significantly more than older children and those with migration background. This could also be confirmed by the results from the linear regression models investigating associations between daily physical activity, television viewing and body composition.

In order to investigate differences in children with long and short sleep duration, three sleep levels were formed. Children with long sleep duration, compared to those with short and medium sleep duration, were grouped in the tertiary sleep level, which was classified as a sleep duration of 10 h and 8 min or more per night. This was – if individually observed – significantly negatively associated with reaching the physical activity guideline of 60 min of MVPA as well as positively associated with daily television viewing of less than one hour. Children’s BMI z-scores based on Cole et al. [39] showed no association with sleep duration, BMI percentiles if classified based on German reference data [49] on the other hand were significantly associated with the tertiary sleep level (data not shown).

Several previous studies have found that sleep duration may play a key role in the development of overweight and obesity [22, 25, 50]. In school-aged children for instance, the risk of being overweight or obese in children who had less than 10 h of sleep on non-school days was far greater than in those who slept more than 10 h per night [28]. In this study however, neither normal weight nor overweight children averaged 10 h of sleep. Still, short sleep duration during childhood was not only associated with present overweight but also with increased body weight in later childhood and adolescence [25, 51] as well as higher BMI values and diabetes risk markers [52]. It could be shown that children with too little sleep at 5 to 6 years of age are more likely to be overweight at 15 years [25] and also children with a short sleep duration at 4 to 5 years showed significantly higher BMI values at 8 and 9 years of age [51]. The latter however, was partially mediated by increased television viewing at 6 to 7 years of age [51].

Apart from such behavioural aspects as television viewing, the suggested reasons for the relationship of sleep and weight status in children vary. Possible approaches assume that sleep duration may be independently associated with children’s metabolic body size phenotype [53] but also that too little sleep can lead either to an increased energy intake or to a reduced metabolic function [22, 54]. Both could have a hormonal explanation, since short sleep duration is associated with increased ghrelin levels (which are known to increase one’s appetite) and a reduced release of the appetite-inhibiting hormone leptin [55–58]. Further has been shown, that sleep deficiency is associated with a stimulation of certain regions in the brain which are sensitive to food stimuli, which points to the assumption that too little sleep might lead to obesity through the selection of high caloric food [54].

Explanations such as the above mentioned are supported by the fact that children who sleep less than 10 h per night consume soft drinks more frequently, eat vegetable less often and consume greater amounts of fried food than children whose sleep duration is longer than 10 h [28, 59]. Further, a long-term study covering a period of 32 years showed that lack of sleep is associated to an increased BMI independently of other behavioural aspects such as media use and socioeconomic status [60].

Behavioural factors however have also been examined in this study; daily television viewing of one hour or less was not associated with children’s sleep duration when analysed in linear regression models but showed significant associations with the tertiary sleep level if analysed in logistic regression models. This is also highlighted by a recent review investigating associations between screen media use and sleep in children and adolescents 5- to 17 years old from different regions around the world [30]. In over 90% of the included studies, more time with screen media was associated with delayed bedtimes and shorter sleep duration among children and adolescents [30]. Among studies associating television viewing with sleep timing and/or quality, over 75% found significant relationships between television watching and too little sleep [30].

This is consistent with most previous research, showing that children with longer periods of television viewing also sleep for shorter periods of time [61–64]. Often this has been attributed to delayed bedtime [65, 66] (which has not been considered in this study) but also to physiological suppression of the sleep-promoting hormone melatonin through the bright light of screens [67]. However, even longitudinal investigations of two-, four, six- and nine-year-olds show, that children with more television use (1.5 h per day or more) at baseline have shorter sleep duration, as well as having a reduction in sleep duration at follow-up with inverse associations of changes in sleep duration [62].

This sample showed comparably low television viewing levels with children spending on average between 45 and 60 min per day watching television. If watching television for one hour or more per day children slept on average 11 min less than those watching less than one hour per day. If they came from families with migration background and low parental education levels television viewing was significantly higher. Once controlled for those factors, for boys, television viewing no longer stayed significantly associated with long sleep duration. For girls on the other hand, low television use was still significantly positively correlated to a tertiary sleep level if controlling for socioeconomic variables.

Similarly, despite the initial significant association between children’s short sleep duration and them reaching the physical activity guideline of 60 min of MVPA on every day of the week [17] in the logistic regression model, once controlled for age, gender, parental education level and migration background, reaching the physical activity guideline stayed no longer significantly associated with a tertiary sleep level. Also, analysing daily MVPA in hours, independent of reaching the recommended 60 min MVPA per day, in linear regressions no association with sleep duration could be found. Yet, there was a difference of 7 min found when comparing more active children with less active children, which also made a difference whether children slept more or less than 10 h. Therefore, children who spent less time in MVPA slept more (than 10 h). Similarly, previous research has shown inconsistent finding regarding children’s physical activity levels. In a recent subjectively assessed sample of 13,000 primary school children in China, children with a sleep duration of 10 h or less were more likely to engage in more moderate and low level physical activity, compared to children sleeping 10 h or more per night [28]. Vigorous physical activity however, was not associated with sleep duration and no physical activity (duration or frequency) was associated with the amount of sleep on weekends [28]. Although other studies have shown relationships between longer sleep duration and increased physical activity [68, 69], a study assessing children slightly younger than the ones in this sample, found that – objectively assessed – the most active children slept 1:02 and 1:40 h less at night compared with the least active children at 5 and 7 years, respectively [70]. Also, a sleep duration of an additional hour in 10- to 12-year-olds has been associated with 20 min less of MVPA during the day [32]; whereas other studies show no relationship between sleep duration and physical activity levels on the following day [24].

Since most studies assess cross-sectional non-experimental data on sleep and physical activity with no meaningful statement on whether physical activity affects sleeping patterns or the other way round, recent research analysed bi-directional associations between sleep duration and MVPA [71]. Lin and colleagues [71] were able to show that if children’s sleep duration was increased by one hour, their time spent in MVPA increased by less than one minute. Further, if MVPA was to predict sleep duration, MVPA was significantly associated with sleep duration; for each one hour increase in MVPA, sleep duration increased by six minutes [71].

In order to investigate and clarify this question further, more longitudinal experimental research with objective measurement methods are needed. However, until then, this research adds valuable insights in associations of children’s sleep duration and behavioural as well as physical factors. In spite of this, these findings should be interpreted with caution since there are some limitations that need to be considered. Since physical activity was estimated on the basis of energy expenditure it could have led to misinterpretations of some children’s intensities and therefore the reported results. Further, the used device recorded children’s activity four times per minute, which may have been not often enough in order to accurately capture every activity. Further, information on television watching was provided by parents in a questionnaire, which is subject to a reporting bias. Moreover, a potential selection bias cannot be ruled out since children, parents and schools participated on a voluntary basis. Furthermore should be noted that the sample – although from a quite widely spread area – is not representative, mainly due to its low overweight and obesity prevalence. A further limitation is the cross-sectional design of this study which does not allow for causal interpretation and the study is relatively small and therefore underpowered, which limits observing potentially significant associations. Additionally, when analysed with logistic regression models, six children (2% of the cohort) who slept more than the recommended 9 to 11 h per night, we included in the highest sleep level, which was not further considered and could also contribute to potential health issues. Despite these limitations, the comprehensive assessment of children’s weight status as well as the consideration of a multiplicity of independent factors should be considered a strength of this study. Further strengths of this study are the objective measurement of body composition and the individual calculation of objectively assessed sleep duration and physical activity which allow a certain reliability of data.

## Conclusions

The here investigated associations of objectively assessed night-time sleep duration with reaching the physical activity guideline of 60 min of MVPA per day and daily television viewing in German primary school children, showed that – although not associated when analysed in linear regression models – both are independently associated with a tertiary sleep level of 10 h and 8 min per night or more. This therefore adds to previously primarily subjectively assessed associations of sleep and risk factors for obesity (related behaviours) with a detailed insight based on objective data. However, more longitudinal and experimental research is needed in order to provide reliable data in order to enable decision making of public health stakeholders, policymakers and practitioners. Interventions trying to decrease children’s BMI and television viewing should also aim at extending children’s night-time sleep and inform parents about the importance of sufficient sleep during childhood in order to pursue a holistic health promotion.

## Abbreviations

BMI z-scores: body mass index z-scores
BMI: body mass index
MVPA: moderate to vigorous physical activity
WHO: World Health Organisation
